# Editorial: Adults with learning difficulties in post-secondary education

**DOI:** 10.3389/fpsyg.2024.1356615

**Published:** 2024-02-01

**Authors:** Miriam Sarid, Orly Lipka, Irit Bar-Kochva

**Affiliations:** ^1^Department of Education and Learning Disabilities, Western Galilee College, Acre, Israel; ^2^Faculty of Education, Department of Learning Disabilities, Edmond J. Safra Brain Research Centre for the Study of Learning Disabilities, University of Haifa, Haifa, Israel; ^3^Department “Teaching, Learning, and Counselling”, German Institute for Adult Education—Leibniz Centre for Lifelong Learning, Bonn, Germany

**Keywords:** learning difficulties, learning disabilities, post-secondary education, adjustment to higher education, ADHD, Autism Spectrum Disorder, reading comprehension, transition to higher education

Education is crucial for employment, individual development, social mobility, and quality of life. Legislation guaranteeing the rights of individuals with disabilities has opened up the possibility of post-secondary education, including higher education, to an increasing number of people with learning difficulties. However, this progress can be accompanied by significant challenges—for adult learners with learning difficulties and for the education system. Challenges for individuals may include persistent difficulties with basic reading, writing, or numeracy skills, difficulties in applying persistent and effective learning behaviors and strategies, and various aspects of social-emotional coping. Challenges at the level of the education system may include insufficient knowledge and awareness of the needs of adults with learning difficulties, as well as limited resources and official regulations for the provision of support to these learners.

Compared to children, adults have traditionally received less attention in scientific research on learning. This Research Topic aimed to increase the theoretical and practical knowledge about adult learners, specifically adults with learning difficulties or with diagnosed learning disabilities, in various types of post-secondary education. Accordingly, the current Research Topic covers a wide range of learning difficulties and diagnosed disorders, from Attention Deficit Hyperactivity Disorder (ADHD) to Autism Spectrum Disorder (ASD), diagnosed learning disabilities (LD), and difficulties with oral and written language processing. In addition, the Research Topic includes a range of contexts for young adults: vocational training, colleges and universities, and the transition from high school to higher education. Finally, the Research Topic relates to challenges at the level of the individual learner as well as at the level of the education system.

In a systematic literature review, Álvarez-Godos et al. examined the support for university students with ADHD. Based on a sample of 24 studies, two types of support were identified. One is support provided by universities, which focuses mainly on academic support, but also personal support and career guidance. The second is intervention programs offered outside of the universities, including cognitive-behavioral therapy, coaching, and mindfulness. Given that students with ADHD may face increasing individual challenges when entering higher education, the authors point to the need to offer a range of actions supporting these students and to examine whether the second type of support, now provided in non-academic settings, could be implemented in universities. The authors concurrently consider the institutional challenges associated with such an implementation.

The *Brief Research Report* by Bar-Kochva et al. presents a study of 32 young adults in vocational training in Germany whose reading comprehension was at primary school level, despite a normal IQ approximation score and having spent between nine and 12 years in school. The study aimed to examine whether the listening comprehension skills of these young adults are impaired as well and whether these skills explain the variance in reading comprehension. The average performance of the group in the listening comprehension tasks was below the level expected by age and education level, yet not all participants showed a deficit in this skill. In addition, listening comprehension, but not word reading, explained a significant amount of variance in reading comprehension. The study indicates several challenges related to this group of adult learners, including their heterogeneity in language skills and the need to consider interventions with oral language components for some learners in this educational context.

The study by Sarid and Lipka addresses the adjustment to higher education of students with ADHD and/or LD in Israeli colleges and universities who had to adapt to higher education in remote learning during the COVID-19 pandemic. The study included four groups of students who were tested in two learning conditions (*N* = 621): 330 students who participated during COVID-19, studying in remote learning (133 with LD/ADHD and 197 without LD/ADHD), and 291 students (65 with LD/ADHD and 226 without LD/ADHD) who were tested before the pandemic, studying in face-to-face mode. The same instruments were used to collect data from all groups. Results indicated that students with LD/ADHD had lower adjustment scores during face-to-face learning and remote learning than the participants without LD/ADHD. Further analyses of subgroups of students who studied during COVID-19 (LD+ADHD, LD, ADHD, and without LD/ADHD) revealed that students with LD+ADHD reported lower academic, emotional, and institutional adjustments as well as lower satisfaction with life while using remote learning than the students without LD/ADHD. ADHD was found to directly predict low satisfaction with life through the mediation of adjustment scores while using remote learning. The study highlights the challenges of students with LD and/or ADHD during distance learning and their need for support in adapting to changes in their learning environment. Recommendations are provided for supporting high-risk LD/ADHD populations during a crisis.

Weis et al. address the need to standardize decisions about academic accommodations for students with disabilities in higher education, such as extended time on exams, distraction-free testing environments, and reading assistance. The authors present the design and validation process of an instrument aimed at systematically assessing specific areas of impairment, planning accommodations, and monitoring the effectiveness of interventions over time, thereby offering a practical tool for accommodations in higher education. The validation process was based on three surveys conducted among a general population of undergraduate students in the United States who were enrolled in studies at post-secondary institutions (*n* = 200, *n* = 325, and *n* = 650) and a sample of 31 professionals. The final instrument covers seven learning domains for higher education students: note-taking, foreign language, social-academic functioning, math, time management, test-taking, and reading.

Finally, Madaus et al. present the results of interviews with the parents of 10 college students who are both academically talented and diagnosed with Autism Spectrum Disorder (ASD). The study aimed to identify the needs of these students in the transition to higher education. Based on the interviews, three key areas of transition support for these students were identified: independent living, self-determination, and enhanced work on executive functioning and social skills. Accordingly, implications and suggestions for professionals were: (1) to facilitate an individualized post-secondary experience; (2) to help students develop and practice social skills; (3) to help students develop and practice executive functioning skills; and (4) to develop awareness of available disability services in college.

Taken together, the studies included in the Research Topic provide insights into the multifaceted challenges of adult learners with learning difficulties and disabilities and the need to reassess institutional resources and support programs accordingly.

## Author contributions

MS: Writing – original draft, Writing – review & editing, Conceptualization, Project administration. OL: Writing – original draft, Writing – review & editing, Conceptualization, Validation. IB-K: Writing – original draft, Writing – review & editing, Conceptualization, Validation.

